# Comparative study of preclinical mouse models of high-grade glioma for nanomedicine research: the importance of reproducing blood-brain barrier heterogeneity

**DOI:** 10.7150/thno.46468

**Published:** 2020-05-15

**Authors:** Caterina Brighi, Lee Reid, Laura A Genovesi, Marija Kojic, Amanda Millar, Zara Bruce, Alison L White, Bryan W Day, Stephen Rose, Andrew K Whittaker, Simon Puttick

**Affiliations:** 1 Australian Institute for Bioengineering and Nanotechnology, The University of Queensland, Brisbane, Australia; 2 ARC Centre of Excellence in Convergent Bio-Nano Science and Technology, The University of Queensland, Brisbane, Australia; 3 Australian e-Health Research Centre, Commonwealth Scientific and Industrial Research Organization, Royal Brisbane and Women's Hospital, Brisbane, Australia; 4 Institute for Molecular Bioscience, The University of Queensland, Brisbane, Australia; 5 Department of Cell and Molecular Biology, QIMR Berghofer Medical Research Institute, Brisbane, Australia; 6 School of Biomedical Sciences, Faculty of Health, Queensland University of Technology, Brisbane, Australia; 7 School of Biomedical Sciences, The University of Queensland, Brisbane, Australia

**Keywords:** High-grade glioma, blood-brain barrier, vascular permeability, nanoparticle-based therapies, preclinical mouse model

## Abstract

The clinical translation of new nanoparticle-based therapies for high-grade glioma (HGG) remains extremely poor. This has partly been due to the lack of suitable preclinical mouse models capable of replicating the complex characteristics of recurrent HGG (rHGG), namely the heterogeneous structural and functional characteristics of the blood-brain barrier (BBB). The goal of this study is to compare the characteristics of the tumor BBB of rHGG with two different mouse models of HGG, the ubiquitously used U87 cell line xenograft model and a patient-derived cell line WK1 xenograft model, in order to assess their suitability for nanomedicine research.

**Method:** Structural MRI was used to assess the extent of BBB opening in mouse models with a fully developed tumor, and dynamic contrast enhanced MRI was used to obtain values of BBB permeability in contrast enhancing tumor. H&E and immunofluorescence staining were used to validate results obtained from the *in vivo* imaging studies.

**Results:** The extent of BBB disruption and permeability in the contrast enhancing tumor was significantly higher in the U87 model than in rHGG. These values in the WK1 model are similar to those of rHGG. The U87 model is not infiltrative, has an entirely abnormal and leaky vasculature and it is not of glial origin. The WK1 model infiltrates into the non-neoplastic brain parenchyma, it has both regions with intact BBB and regions with leaky BBB and remains of glial origin.

**Conclusion:** The WK1 mouse model more accurately reproduces the extent of BBB disruption, the level of BBB permeability and the histopathological characteristics found in rHGG patients than the U87 mouse model, and is therefore a more clinically relevant model for preclinical evaluations of emerging nanoparticle-based therapies for HGG.

## Introduction

The concept of engineering nanoparticles as therapeutic carriers for the treatment of cancer remains the subject of significant research effort. As the field has matured, research effort has shifted from materials design and synthesis to evaluation of therapeutic efficacy *in vivo* and there is a growing interest in the application of the paradigm in more difficult to treat cancers such as high-grade glioma (HGG). HGG is one of the most aggressive primary brain tumors with very poor prognosis. Current standard of care involves maximal surgical resection followed by combined temozolomide chemotherapy and radiotherapy, after which median survival is only 15 months 1. Unlike many non-central nervous system cancers there has been very little improvement in prognosis for HGG patients 2. One of the main challenges currently limiting therapeutic improvements in the treatment of HGG is the inability to achieve an efficient systemic delivery of therapeutics to tumor cells. This is limited by several factors including non-specific binding of the targeting drug to normal tissues; inability of the targeting agent to cross the blood-brain barrier (BBB) and reach the target expressed either on tumor cells or in the tumor microenvironment; dose-related toxicity to surrounding normal brain tissue; reduced targeting agent specificity to tumor cells due to the protein corona; kinetics of internalisation of the targeting agent; poor drug distribution in the tumor microenvironment due to interstitial fluid pressure; and drug efflux pump mechanisms limiting effective drug access to brain tumor cells. As well-described in a number of recent reviews 3-6, each of these factors has a high-level of complexity and, as such, we have focused this investigation on the characteristics of the BBB in HGG as this remains a central challenge affecting the delivery of systemic drugs to the tumor 7. The BBB is a specialized system comprising microvascular endothelial cells, pericytes, astrocytes, tight junctions, neurons and the basement membrane, acting as a physical barrier protecting the brain from toxic substances circulating in the blood stream, including drugs 7. We have recently highlighted how the structural heterogeneity and the impaired functionality of the BBB in HGG are primarily responsible for an inefficient delivery of systemic drugs to the entire population of tumor cells 8.

Increased delivery of drug payloads to tumor tissue is a central pillar of nanomedicine and over the past 10 years, the attention of the nanomedicine community has turned to increased delivery of drug payloads to HGG. Nanoparticle constructs made of biodegradable and nontoxic biopolymers are valuable platforms with a great potential to enhance therapeutic effects of chemotherapeutic drugs in HGG patients. Through careful design, passive and active targeting mechanisms of nanoparticle drug carriers can potentially be used to achieve increased drug uptake into tumor cells 9-13. Moreover, nanoparticle constructs can act as theranostic agents, combining imaging agent and drug transport properties, thereby allowing for the *in vivo* assessment of drug accumulation into the tumor 9,14. These characteristics make nanomedicine an attractive therapeutic approach to tackle the complexity of HGG. As research efforts in the field of nanomedicine for the treatment of HGG increase, it is critical that cross-disciplinary collaboration is actively pursued and that the design of new studies is guided by strong clinical relevance to ensure the highest potential for translation 15-18.

The use of mouse models for preclinical evaluation of new therapies is central to the translational process; however, the suitability of preclinical mouse models of HGG is a controversial topic 19. On one hand, the need for preclinical models to advance the understanding of mechanisms driving tumor development and progression is clear. On the other hand, the clinical relevance of many models as a surrogate for HGG patients to evaluate new therapies is tenuous, as is reflected in the limited success with which new therapies for HGG are translated into the clinic 19,20. There is now an ever-growing list of novel treatments that showed high efficacy in preclinical mouse models, but failed to have the same positive outcome in phase II/III clinical trials 19,21. This has, in part, been attributed to the lack of mouse models able to fully recapitulate the complexity of HGG 22. In particular, the heterogeneous structure and functional characteristics of the BBB in HGG tumors are key factors often overlooked in studies assessing targeted delivery of new therapeutics and is a particularly pertinent concern for the investigation of nanoparticle delivery systems 3,23. A recent review of the clinical literature by Sarkaria *et al*. highlighted that the majority of HGG is characterized by significant heterogeneity in the permeability of the BBB across the tumor and that regions of the tumor with an intact BBB are often populated by infiltrating tumor cells 24,25. These pockets of infiltration are arguably one of the main causes for the high frequency of tumor recurrence, as they are very difficult to treat, being largely unresectable by surgical means and very difficult to target with chemotherapy and radiotherapy 24,26. In order to address this clinical challenge, two critical steps towards improving the rate of translation of novel nanomedicines need to be taken. First, the development of nanoparticle delivery systems specifically targeting these infiltrating tumor cells. Then, the evaluation of these systems in preclinical mouse models able to reproduce the BBB heterogeneity of recurrent HGG.

Current mouse models of HGG can be broadly classified in three categories: xenografts, genetically engineered mouse models and chemically induced syngeneic murine models 27. Considering the advantages and pitfalls of each of these models, which have been extensively reviewed by Huszthy *et al*., is essential for the choice of an appropriate model for the specific purpose of each study 20. When considering clinical relevance of mouse models for HGG, it is important to distinguish between subcutaneous xenograft models (HGG cells grafted subcutaneously in the flank of a mouse) and orthotopic xenograft models (HGG cells grafted intracranially into the brain of a mouse). Despite the many clear arguments that a brain tumor grown subcutaneously on the flank of a mouse will not reproduce the characteristics of the BBB in HGG, these models continue to dominate the literature. As such, for the remainder of this work we only consider intracranial xenograft models as relevant models from the perspective of representing the challenges of the BBB.

**Figure 1:**
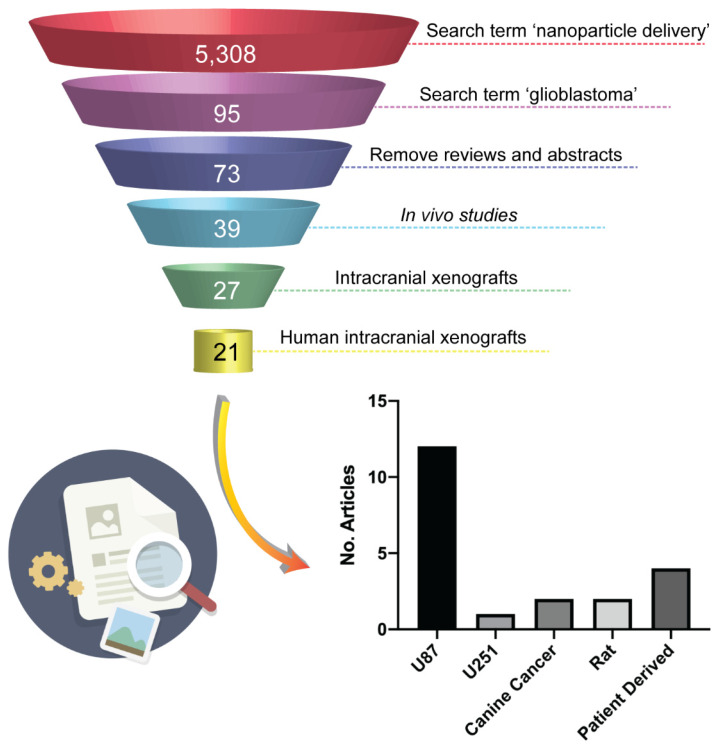
** Literature search of animal models in preclinical nanomedicine research.** The illustration shows the filtration process used in the search performed with Web of Science to identify journal articles addressing preclinical evaluation of nanoparticle-based therapies in human intracranial xenografts models of HGG. The chart shows that more than 50% of the 21 relevant papers emerged from the search report the use intracranial xenograft mouse models derived from the U87 cell-line.

Intracranial xenograft models formed by injection of a bolus of cells into the brain (cell-line xenografts - CLX) have been a central pillar of preclinical investigations of new therapeutics for many years 20. Examples of commercially available and ubiquitously used HGG cell lines for xenograft models are U87, U251, T98G and A172 27. While these models have the advantages of high growth rates and high engraftment success, good reproducibility and reliable tumor growth and progression, they often develop neoplasms with a lack of infiltration and necrosis, and highly upregulated angiogenesis 27-29. In particular, the lack of single-cell invasion, the well-defined border of the tumor and the highly angiogenic characteristics of neoplasms derived from U87 cell lines lead to tumors that fail to faithfully reproduce the vascular characteristics of the majority of HGG patients 20,28, namely, a partially intact BBB. This phenotypic deviation from a patient's tumor characteristics has important implications for their use in the assessment of delivery of new systemic therapies, but in particular of nanoparticle-based therapies. In the field of nanomedicine research, there is a large bias towards the use of models derived from U87 cell lines in studies assessing nanoparticle delivery systems. This could potentially be attributed to the observation that the large BBB disruption in these tumor models results in a high tumor enhanced permeability and retention (EPR) effect, which leads to an increase in “positive” results of nanoparticles targeting efficiency. To illustrate the prevalence of these models in current literature we conducted a short review within the field of nanomedicine. We searched Clarivate Analytics Web of Science using the search terms 'nanoparticle delivery' over the past two years (2018 and 2019) and conducted a search within the results for 'glioblastoma' which yielded 95 papers that were the subject of our review. Removing review articles, conference abstracts, articles that were not related to HGG and articles with no *in vitro* or *in vivo* data resulted in 73 papers describing therapeutic efficacy of a nanoparticle delivery system in HGG. 34 papers presented only *in vitro* data and 12 papers reported data from subcutaneous mouse models and were discounted from further analysis. 6 papers reported the use of the syngeneic GL261 mouse model which, not being a human cell line, is not the subject of this work and were discounted from further analysis. Of the remaining 21 articles, 12 papers reported data using intracranial xenograft models derived from implantation of U87 cells (**Figure 1**) representing over 50% of *in vivo* orthotopic literature in the nanomedicine field. As such, we have focused our current study on comparing the characteristics of the BBB in intracranial xenograft mouse models derived from the U87 cell line and a patient-derived cell line from our lab with patients with recurrent HGG.

We have generated a curated panel of patient-derived, low-passage, serum-free HGG cell lines that accurately recapitulate genotypes and phenotypes of HGG in intracranial xenografts 30,31. In this work, for the first time, we conducted a head to head comparison of the extent of BBB disruption in the U87 CLX mouse model, one of our patient-derived cell line models termed WK1 and patients with recurrent HGG (rHGG). Using contrast enhanced (CE) MRI we show that our WK1 mouse model is better able to recapitulate the extent of BBB disruption seen in patients than the U87 model. Furthermore, using semi-quantitative dynamic contrast enhanced MRI (DCE MRI) and histology, we show that even in regions of contrast enhancement, the U87 model poorly reproduces the clinical reality of the tumor BBB physiology, whereas our WK1 model shows far better correlation.

## Method

### Experimental design

All animal experiments were approved by The University of Queensland Animal Ethics Committee and QIMR Berghofer Animal Ethics Committee and adhered to the experimental animal care guidelines. A total of 30 NOD/SCID tumor-bearing mice were used for this study. Animals were divided into two groups, a U87 group of 12 mice and a WK1 group of 18 mice. Before each procedure and imaging session, the mice were anesthetized with 2% isoflurane (Isothesia^®^ NXT; Henrt Schein Animal Health, USA) in air at a flow rate of 2 L/min.

Structural MRI was used in this study to assess tumor size and extent of BBB opening. T_2_-weighted MRI images (T2) were acquired to characterize the tumor volume and surrounding regions of edema. CE T_1_-weighted MRI images (T1-CE) were used to determine the extent of BBB disruption. DCE imaging was used to characterize the permeability of the BBB in CE areas of the tumor. Body temperature was maintained throughout the imaging session with a heated water bed and breathing rate was monitored at all times with a respiratory pillow.

For both mouse models, tumor cells were implanted in the same location of the brain when the mice were 6-weeks old. Tumor development was assessed *via* T2 imaging and signs of health impairment were monitored for four months post-implantation. Mice were enrolled in the experiment when the tumor size reached a size of 200 ± 100 mm^3^ in one plane and the mice showed signs of sever health impairment. The U87 groups were enrolled in the experiment on average 6-8 weeks post implantation and the WK1 xenograft on average 12-16 weeks post implantation. The complete imaging protocol consisted of the following MRI scans: T2, T_1_ maps, a DCE sequence and a T1-CE. Following MRI acquisitions, all the mice were euthanized by cardiac perfusion with ice cold phosphate buffered saline (PBS) followed by 4% paraformaldehyde (PFA). The brains were excised and stored in 4% PFA at 4 °C, washed twice with 0.1% NaN_3_ in PBS after 24 and 48 h, and then stored in 70% ethanol at 4 °C until embedding in paraffin. Finally, histology was performed on the paraffin-embedded brains.

### Tumor models

#### WK1 patient-derived xenograft

The patient-derived cell line tumor model used in this study was generated by orthotopic injection of WK1 cells. The WK1 cell line was derived from a 77-year old man with right parieto-occipital glioblastoma, prior to him receiving chemotherapy or radiotherapy. Cells were isolated from surgical aspirate collected during resection as reported previously 31. Tumor tissue was collected as part of a study approved by the Human Ethics Committees of the QIMR Berghofer Medical Research Institute and the Royal Brisbane and Women's Hospital with full patient consent. Cells were grown as an adherent culture in Matrigel-coated tissue culture flasks in RHB-A stem cell culture medium (StemCell Inc, Newark, CA) containing 100 U/mL penicillin and 100 μg/ml streptomycin and supplemented with 20 ng/ml epidermal growth factor and 10 ng/ml fibroblast growth factor (StemCell Inc, Newark, CA). All cells were cultured in 5% CO_2_/95% humidified air atmosphere at 37 ºC and used on achieving 70-80% confluence. Cells (100,000 in 2 μl of PBS) were injected into the right striatum of 6-week old female NOD/SCID mice using a stereotaxic frame under isoflurane anesthesia. Cells were injected at a depth of 3 mm, 1.6 mm rostral and 0.8 mm lateral to the bregma. WK1 was obtained from a characterized glioblastoma patient-derived cell line resource, the data for which is publicly available from Q-Cell website and in the following references 30-33. In our hands, the success rate of tumor formation is approximately 90%. The median survival of WK1 xenograft models is 150±2 days, as previously reported by Stringer *et al*. 30.

#### U87 cell-line xenograft

The CLX tumor model used in this study was generated by orthotopic injection of U87 cells. All cells were cultured in 5% CO_2_/95% humidified air atmosphere at 37 ºC and used on achieving 70-80% confluence. Cells (100,000 in 2 μL of PBS) were injected into the right striatum of 6-week old female NOD/SCID mice using a stereotaxic frame under isoflurane anesthesia. Cells were injected at a depth of 3 mm, 1.6 mm rostral and 0.8 mm lateral to the bregma.

### MRI

MR images were acquired on a Bruker 7T Clinscan interfaced with a Siemens spectrometer running Numaris 4 VB17 using a 23 mm mouse head volume coil. Animals were anesthetized and a catheter pre-loaded with the gadolinium contrast agent (CA) (gadobutrol, 0.1 mmol/kg, Gadovist^®^ 1.0; Bayer) was placed in the tail vein. Imaging sequences included a T2 (resolution 0.078 × 0.078 × 0.700 mm^3^; TR/TE 2750/45 ms/ms; flip angle 180°), T_1_ maps (resolution 0.195 × 0.195 × 0.850 mm^3^; TR/TE 12/0.93 ms/ms; flip angles 10, 15, 20, 25, 30°), DCE (resolution 0.195 × 0.195 × 0.850 mm^3^; TR/TE 12/0.93 ms/ms; flip angle 12°), and T1-CE (resolution 0.117 × 0.117 × 0.120 mm^3^; TR/TE 12/1.78 ms/ms; flip angle 21°). DCE MRI images were acquired before, during and after i.v. injection of the gadolinium bolus (40 μl at a rate of 10 μl/s).

### H&E, Immunofluorescence and Microscopy

Paraffin embedded mouse brains were cut into 7 μm thick coronal sections with a Rotary Microtome HM 355 S (Microm International). Hematoxylin and eosin (H&E) staining was used for histopathological examination. Following deparaffinization, slides were stained in Hematoxylin (Sigma Aldrich) for 3 min and the excess of hematoxylin was removed by short immersion of slides in 1% HCl, followed by 0.1% LiCO^3^. Next, slides were stained in Eosin Y solution (Sigma Aldrich) for 30 s and dehydrated by using 70, 90, and 100% ethanol for 30 s each, followed by xylene for 10 min. Slides were mounted with Entellan mounting medium (ProSciTech) and dried for 2 h. Images were captured using Aperio Bright field XT slide scanner (ScanScope® XT). Expression of glial fibrillary acidic protein (GFAP), plasmalemma vesicle associated protein 1 (PLVAP) and cluster of differentiation (CD31) were assessed by immunofluorescence staining as per standard protocol. Heat-activated, tris-based pH 9.0 antigen retrieval was performed, with horse serum (4% in bovine serum albumin) and M.O.M blocking solution (BMK-2202; Vector Laboratories) used to prevent non-specific binding of primary antibodies. The primary antibodies used were mouse anti-GFAP (MAB360; 1:100 dilution; Merck), rabbit anti-CD31 (#28364; 1:20 dilution; Abcam) and rat anti-PLVAP (#553849, 10 μg/ml, 1:50 dilution; BD Biosciences). Incubation with an AF488 donkey anti-rat, AF594-labelled donkey anti-rabbit and AF488-labelled donkey anti-mouse (1:250 dilution; Invitrogen) and counterstained with DAPI (Sigma Aldrich). Images were captured using Axiovert 200 inverted confocal microscope with LSM 710 scanner (Carl Zeiss Pty Ltd) as Z-stacks and presented as the sum of the Z-stack projection. Whole-brain images were acquired as tiled image stacks. Image processing was performed by using ImageScope and ImageJ software.

### rHGG patient dataset

The rHGG patient dataset was downloaded from the RIDER NEURO MRI project 34, stored in The Cancer Imaging Archive 35. This consisted of MR imaging from 16 patients with recurrent glioblastoma acquired at 1.5 T. Imaging sequences included a T_2_ 3D FLAIR (resolution 1.0 × 1.0 × 1.0 mm^3^; TR/TE 6000/353 ms/ms; flip angle 180°), T_1_ maps Multi-flip 3D FLASH images (resolution 1.0 × 1.0 × 5.0 mm^3^; TR/TE 4.43/2.10 ms/ms; flip angles 5, 10, 15, 20, 25, 30°), T1-CE sagittal 3D flash images (resolution 1.0 × 1.0 × 1.0 mm^3^; TR/TE 8.6/14.1 ms/ms; flip angle 20°), and DCE 3D FLASH images (resolution 1.0 × 1.0 × 5.0 mm^3^; TR/TE 3.8/1.8 ms/ms; flip angle 25°). DCE sequences were obtained during the intravenous injection of 0.1mmol/kg of Magnevist^®^ at 3ccs/s, started 24 s after the scan had begun.

### Image Analysis

#### Structural MRI image analysis

**Figure 2:**
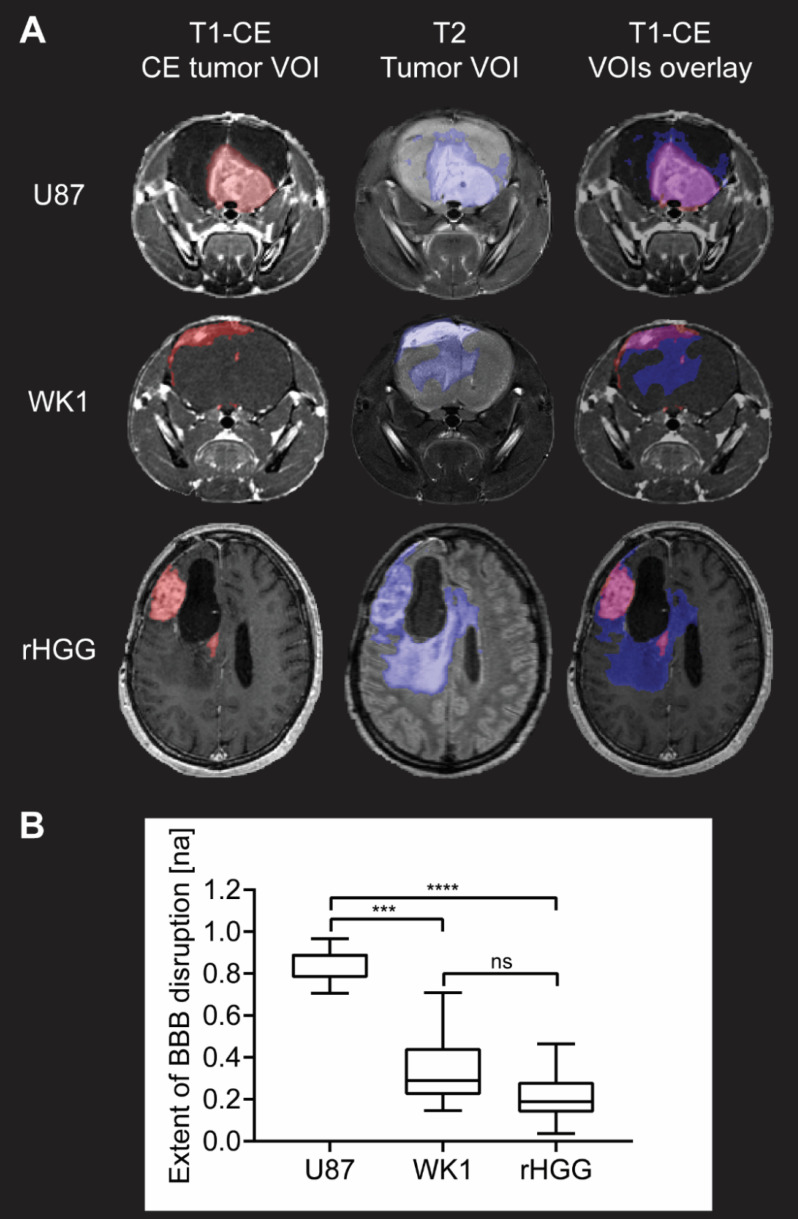
** Evaluation of extent of BBB disruption from MR images. A**. T1-CE with defined CE tumor VOI (red), T2 with defined tumor VOI (blue) and T1-CE with overlay of CE tumor VOI (red) and tumor VOI (blue) for one example subject from each of the three groups. **B**. Graph of quantitative analysis of extent of BBB disruption in the tumor in the three groups. The graph shows median values of extent of BBB disruption with whiskers representing minimum and maximum values in the group, * p < 0.05, ** p < 0.01, *** p < 0.001, **** p < 0.0001, ns = no significant difference.

DICOM images were converted into NIFTI format using a combination of dcm2niix and MRtrix3 36,37. T2 and DCE images were then rigid registered using rigid transformations only to the post contrast T1-CE followed by linear resampling into this space using ANTS for the mice dataset and using FSL FLIRT for the patient dataset 38-40. Binary masks of the tumor volume of interest (VOI) and CE tumor VOI were manually delineated from the T2 and T1-CE images, respectively, using a semi-automatic active contour segmentation tool (ITK-SNAP 41; **Figure 2A**).

Statistics for each VOI, including volume and mean intensity values, were calculated using FSLSTATS software 42. The ratio of the CE tumor VOI volume to the T_2_-weighted tumor VOI volume represents the proportion of the entire tumor region that has a disrupted BBB and will be referred to as the extent of BBB disruption from this point. This ratio was used to calculate and compare the extent of BBB disruption between the two tumor models and the patient dataset.

#### DCE sequence image analysis

T_1_ maps and DCE sequences were imported into the Nordic-ICE^®^ (NordicNeuroLab, Bergen, Norway) software package and maps of K_trans_, the rate constant defining the transport of CA from the intravascular space to the extravascular space 43,44, and K_ep_, the rate constant defining the transport of CA from the extravascular space to the intravascular space, were generated using the extended Tofts' model 43,45. Image pre-processing consisted of noise correction, motion artefact correction and baseline T_1_ correction using acquired T_1_ maps. The signal was normalized by an arterial input function (AIF), obtained from the selection of 10-15 voxels in the occipital artery in the patient dataset and in arteries with good signal-to-noise ratio in the mice dataset. Selected AIFs and generated K_trans_ maps are shown in **Figures S1**, **S2** and** S3**. Mean voxel-wise K_trans_ values within the CE tumor (as defined by the CE tumor VOI mask) were calculated then averaged across all voxels with non-zero signal intensity. Zero-signal intensity voxels were removed to avoid including voxels where the K_trans_ model had failed to fit. This is a normal occurrence that can be caused by T_2_* effects and image noise that can cause an underestimation of the magnitude of the peak of the AIF in some voxels, particularly when working with high resolution images or high magnetic fields 46,47. Values of mean K_trans_ and mean K_ep_ in the CE tumor VOI for each subject are collected in **Table S1**.

### Statistical Analyses

Statistical analyses were performed using GraphPad Prism 7 Software. Dunn's multiple comparison test after Kruskal-Wallis one-way non-parametric test, α=0.05 was used to determine significance in the comparison of mean values of extent of BBB disruption between each pair of groups, and significance in the comparison of mean values of BBB permeability (mean K_trans_) in the CE tumor between the three groups (n = 12 for the U87 group, n = 18 for the WK1 group and n = 16 for the patient rHGG group).

## Results and Discussion

### U87 model does not recapitulate BBB heterogeneity of recurrent HGG patients

Structural MRI analysis reveals extensive BBB disruption in the U87 mouse model, whilst the CE tumor volume corresponds only to a small proportion of the entire tumor in the WK1 tumor model (**Figure 2A**). As shown by the overlay of the T1-CE and the T2, this imaging phenotype of the WK1 mouse model is very similar to that seen in rHGG patients, where a large part of the T2-hyperintense tumor lesion is not contrast enhancing. These qualitative observations are mirrored in the quantitative analysis of the extent of BBB disruption (**Figure 2B**). The mean extent of BBB disruption is substantially higher in the U87 model (0.8511; p<0.0001) than in the patient dataset (0.2249; **Figure 2**). This highlights that the U87 model does not accurately reproduce the vasculature characteristics of rHGG patients. By contrast, the WK1 model demonstrates mean BBB disruption extent of 0.3307, significantly lower than the U87 dataset (p=0.0004), but not significantly different to the rHGG dataset (p=0.2813). This suggests that this model is more representative of the heterogeneity of the BBB characteristics observed in most rHGG patients. As such, this model would be expected to deliver more reliable predictions of the efficiency of delivery of systemic therapies.

### U87 model has an excessively higher BBB permeability than recurrent HGG patients

**Figure 3:**
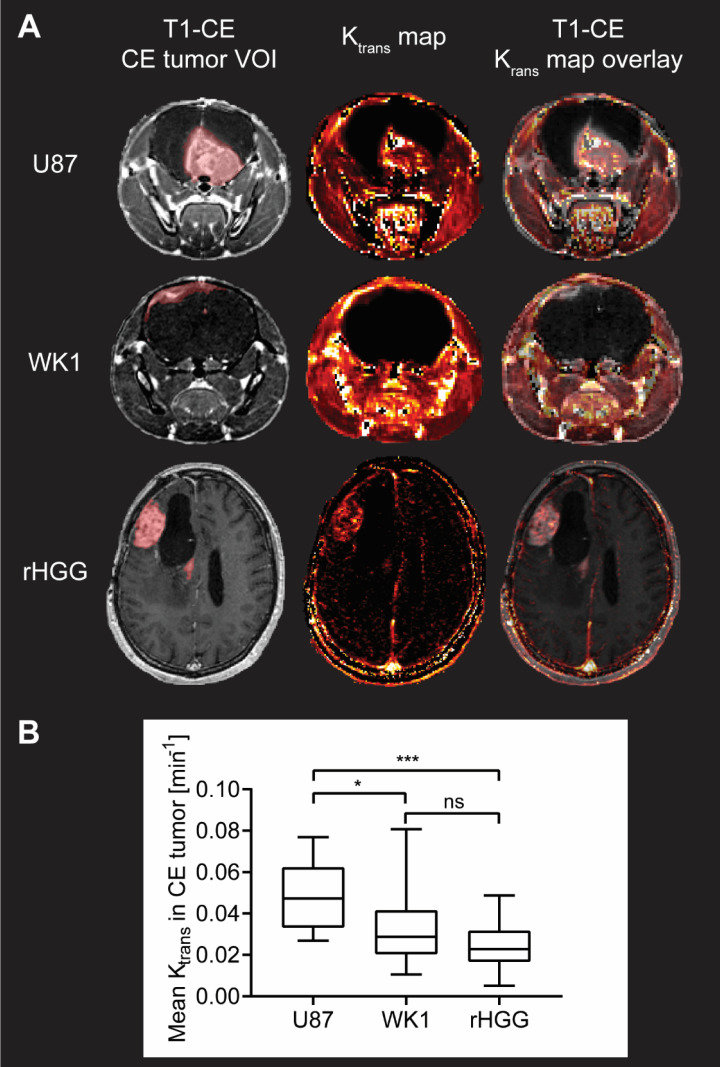
** Evaluation of BBB permeability in CE tumor from DCE MRI analysis. A**. T1-CE with defined CE tumor VOI (red), K_trans_ map generated from kinetic modelling of DCE MRI data and T1-CE with overlay of K_trans_ map for one example subject from each of the three groups. **B**. Graph of quantitative analysis of BBB permeability in the CE tumor in the three groups. The graph shows median values of mean K_trans_ in the CE tumor VOI with whiskers representing minimum and maximum values in the group, * p < 0.05, ** p < 0.01, *** p < 0.001, **** p < 0.0001, ns = no significant difference.

BBB permeability in the CE tumor is assessed by measuring values of K_trans_. **Figure 3A** shows examples of K_trans_ maps generated by voxel-wise modelling of DCE MRI data; images from all subjects along with AIF used for modelling are shown in **Figures S1**, **S2** and **S3**.

Quantitative analysis of the median values of K_trans_ in the CE tumor VOI is summarized in **Figure 3B**. Mean values of K_trans_ in the CE tumor are 0.04812 min^-1^ for U87, which is consistent with previously reported values of K_trans_ in mouse models derived from the U87 cell line 48-51, but significantly higher than both the WK1 xenografts (0.03142 min^-1^, p=0.0154) and the rHGG patient dataset (0.02480 min^-1^, p=0.0008). Such excessive permeability of the BBB in the CE tumor in the U87 xenografts further suggests that this mouse model poorly represents the physiological properties of rHGG patients. Conversely, there is no significant difference between mean values of BBB permeability in the CE tumor between the WK1 model and rHGG patients (p=0.9091). This suggests that WK1 serves as a much more robust preclinical model replicating the BBB physiology in the CE tumor of rHGG patients and is a more accurate platform to assess extravasation and tumor uptake of systemic therapies.

### Histopathology and immunofluorescence analysis of two mouse models

**Figure 4:**
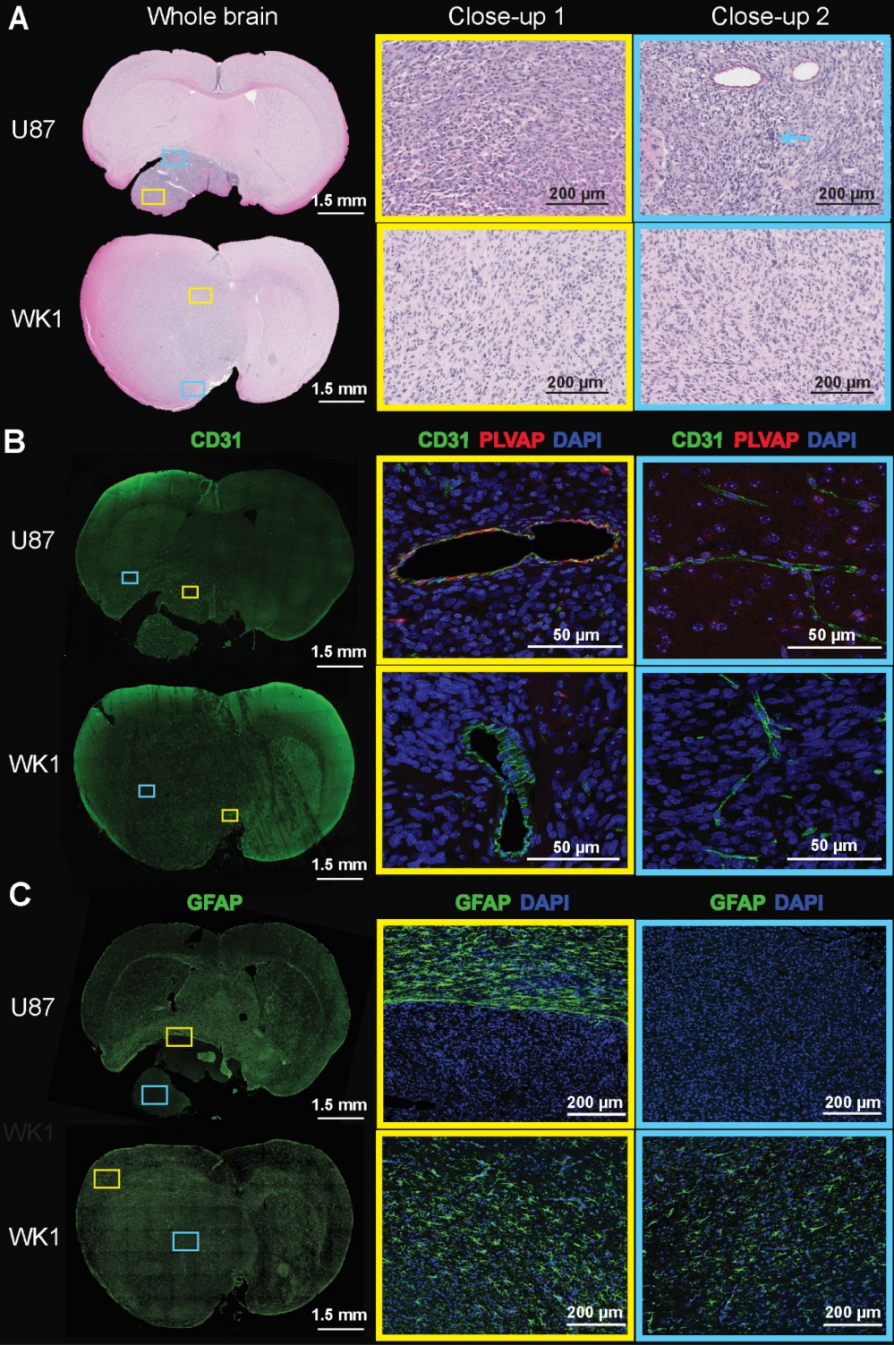
** Images of H&E and immunofluorescence stained brain sections.** The images show **A.** H&E, **B.** CD31/PLVAP/DAPI and **C.** GFAP staining of whole brain sections, and magnification of two areas (close-up 1 - yellow rectangle, close-up 2 - blue rectangle) of the U87 and WK1 mouse models.

Histopathological examination of the U87 xenograft is consistent with previous reports, characterized by a well demarked, non-infiltrating nodular tumor mass 28,52. This mass comprises closely packed round to polygonal cells with abundant intensely eosinophilic cytoplasm (yellow box) and frequent large multinuclear agglomerates (blue arrow), lacking regions of pseudopalisading necrosis (blue box) (**Figure 4A** and**Figure S4A**, top). By contrast, the WK1 xenograft is characterized by a meningeal expanding tumor mass in the superior part of the right lobe, and a large area of infiltrating tumor corresponding to non-CE tumor. The tumor mass is characterized by elongated glioma cells (yellow box) and regions of pseudopalisading necrosis (blue box), in accordance with what previously reported on another patient-derived xenograft mouse models of HGG 52. Given human HGGs are characterized by a high degree of cellular density with pleomorphism, regions of pseudopalisading necrosis and tumor cell invasion in non-neoplastic brain parenchyma 52,53, our findings confirm that the WK1 model more closely recapitulates the histopathological characteristics of the majority of rHGG patients than the U87 mouse model.

The differences in the permeability of vasculature between these models prompted us to investigate whether this corresponded to any vascular characteristics on a molecular level. Examination of immunofluorescence staining of brain sections with the vasculature endothelial marker CD31 reveals that differences in structural and subcellular vascular features correlate to differences in contrast enhancement of the tumor. The U87 xenograft is characterized by the presence of several large, dilated vessels throughout the entire tumor mass (**Figure 4B** and**Figure S4B**, yellow box) and regular microvasculature typical of the normal BBB in the surrounding normal brain (**Figure 4B** and**Figure S4B**, blue box), as previously reported 28,52. Vessels of similar structure are observed in infiltrating tumor tissue of the WK1 xenograft **(Figure 4B** and**Figure S4B**, blue box) corresponding to the non-CE tumor on the T1-CE, whilst large, dilated vessels (**Figure 4B** and**Figure S4B**, yellow box) are observed in tumor tissue corresponding to the CE tumor region on the T1-CE. Once again, the structural vascular characteristics of the WK1 mouse model are much more similar than those of the U87 model to those of human HGGs, which are characterized by both regions of microvasculature proliferation and regions of infiltrating tumor with an intact BBB 52,53. Interestingly, a number of vessels within tumor tissue of the U87 xenograft also express PLVAP. PLVAP is a key structural component of capillary fenestrations associated with trans-endothelial transport that is not normally found in mature endothelium of the brain 54. Given PLVAP expression is induced in brain tumors and associated with breakdown of the BBB 55-57, it is highly likely that PLVAP-mediated trans-endothelial transport of Gadovist contributes to the higher levels of K_trans_ observed in the U87 xenograft than WK1 xenograft.

Finally, immunofluorescence for astrogliosis marker GFAP (**Figure 4C** and**Figure S4C**) shows that the U87 xenograft displays a complete lack of GFAP expression within the tumor mass (blue box), in accordance to previous studies 28,52, with a compact rim of reactive astrogliosis in the neighboring brain region confining the neoplastic structures (yellow box). On the contrary, the WK1 xenograft is characterized by a moderate and non-uniform expression of GFAP both in the infiltrating tumor tissue (blue box) and in the meningeal tumor tissue (yellow box), similar to that observed in the majority of human HGGs 52,53. These results confirm that the WK1 xenograft has retained its glial origin character, whilst the U87 CLX no longer retains the original tumors astrocyte identity.

## Conclusions

In this study we have highlighted significant differences in the extent of BBB opening and BBB permeability between two mouse models of HGG and patient rHGGs, and discussed how these are critical in the choice of a suitable mouse model for the evaluation of the targeting efficiency of novel nanoparticle-based therapies. With CE MRI we demonstrated that the extent of BBB opening in the entire tumor is much larger in the HGG model derived from U87 cells than in the WK1 model, and that the extent of BBB opening present in the WK1 model much more closely resembles the imaging phenotype of the majority of rHGG patients 24. Furthermore, our measurements of median K_trans_ showed that the U87 tumor model has a significantly higher BBB permeability in CE tumor regions than human rHGGs, which has crucial implications for the use of this model in preclinical experiments evaluating delivery efficacy of novel nanoparticle-based therapies. We would strongly argue that the testing of new nanoparticle delivery systems in a mouse model of HGG with an excessively abnormal and dysfunctional BBB is not informative of whether the delivery of that therapy would be effective in patients. Finally, we confirmed that the histopathological characteristics of the WK1 mouse model, but not the U87 mouse model, faithfully reproduce the characteristics of the majority of rHGG patients. With these points in mind, we believe that low-passage, serum-free cell lines derived from patients xenografts, such as the WK1 cell line, provide a better platform for more valuable preclinical models that can be used to more realistically assess the uptake and efficacy of new nanoparticle-based therapies in rHGG.

## Supplementary Material

Supplementary figures and tables.

## Abbreviations

BBB: blood-brain barrier
CA: contrast agent
CE: contrast enhanced
CLX: cell-line xenograft
DCE: dynamic contrast enhanced
GFAP: glial fibrillary acidic protein
HGG: high-grade glioma
H&E: hematoxylin and eosin
K_ep_: rate constant of transport of contrast agent from the extravascular space into the vasculature
K_trans_: rate constant of transport of contrast agent from the vasculature into the brain
MRI: magnetic resonance imaging
PBS: phosphate buffered saline
PFA: paraformaldehyde
PLVAP: plasmalemma vesicle associated protein 1
rHGG: recurrent high-grade glioma patients
T1-CE: contrast enhanced T_1_-weighted MRI image
T2: T_2_-weighted MRI image
VOI: volume of interest
