# Supplementary material for: Comparative study of preclinical mouse models of high-grade glioma for nanomedicine research: the importance of reproducing blood-brain barrier heterogeneity
Source: Theranostics. 2020 May 15;10(14):6361–71. doi: 10.7150/thno.46468 (PMC7255036; doi:10.7150/thno.46468)
Supplement: Supplementary file 1 — Supplementary figures and tables. [file thnov10p6361s1.pdf]

Supplementary Information

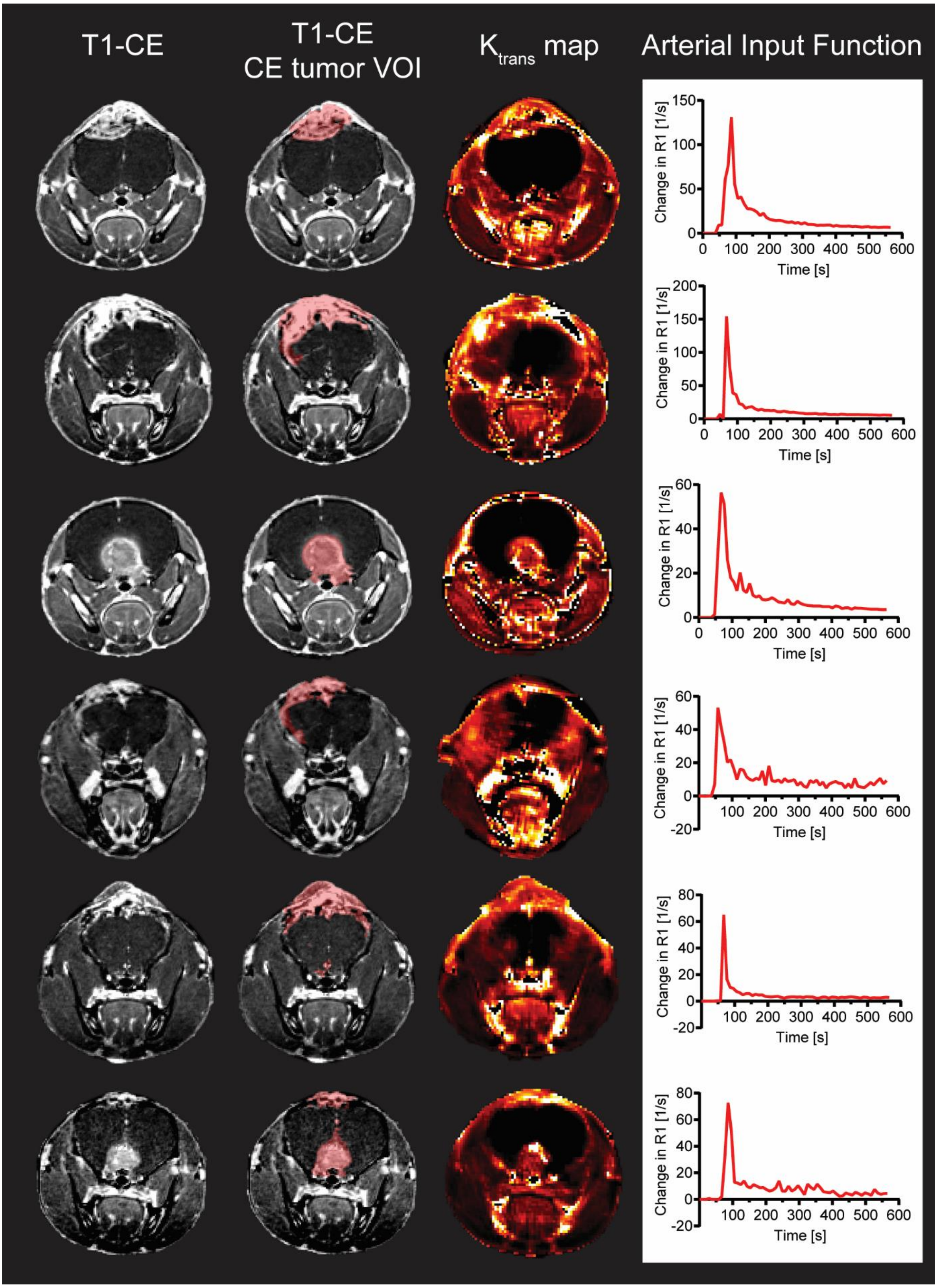

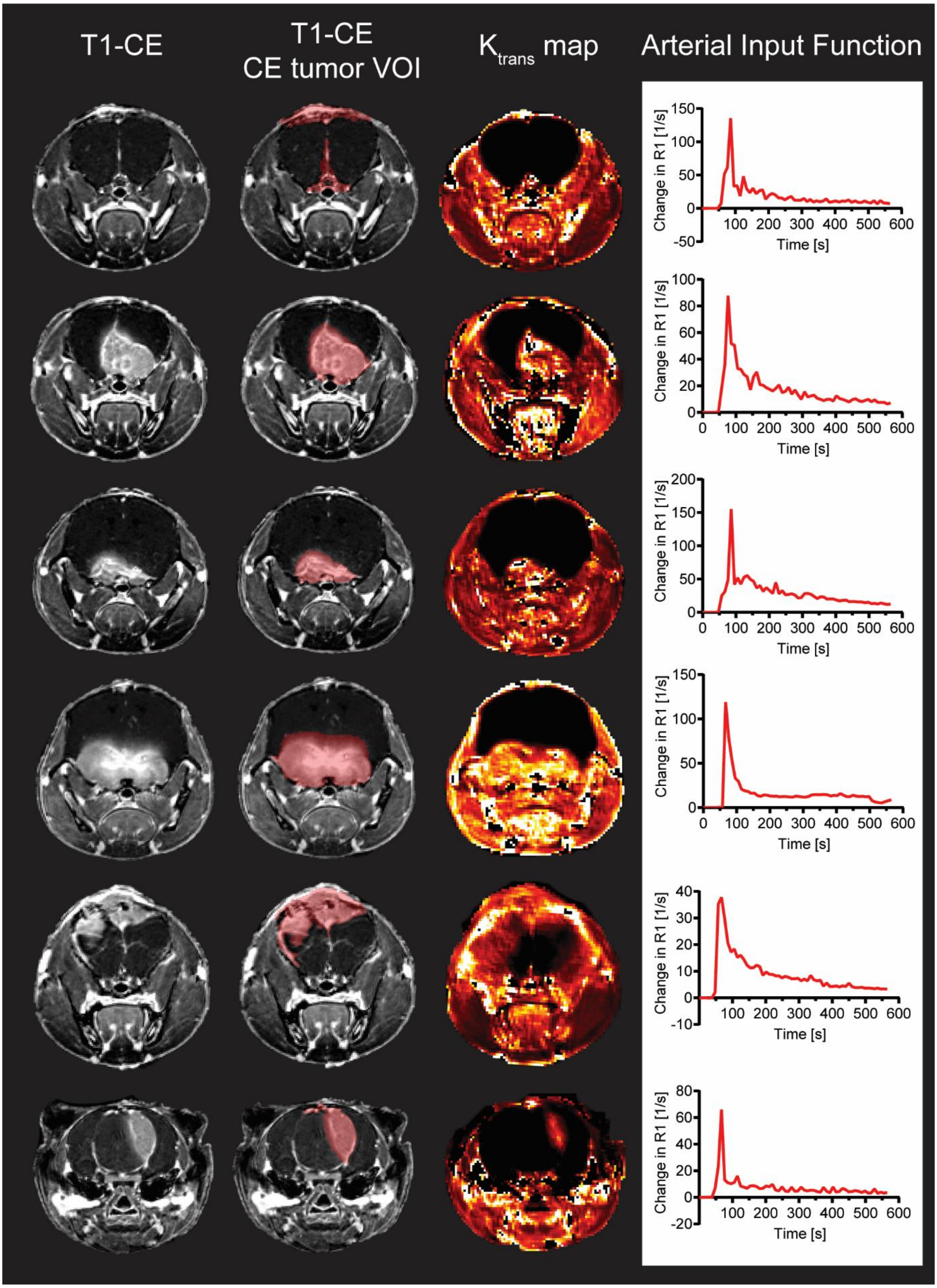

Figure S1. *DCE MRI modelling for K_{trans} maps generation in U87 mouse model*. L-R: T1-CE, T1-CE with defined CE tumor VOI (red), K_{trans} map generated from kinetic modelling of DCE MRI data and arterial input function (AIF) selected for the generation for the K_{trans} map.

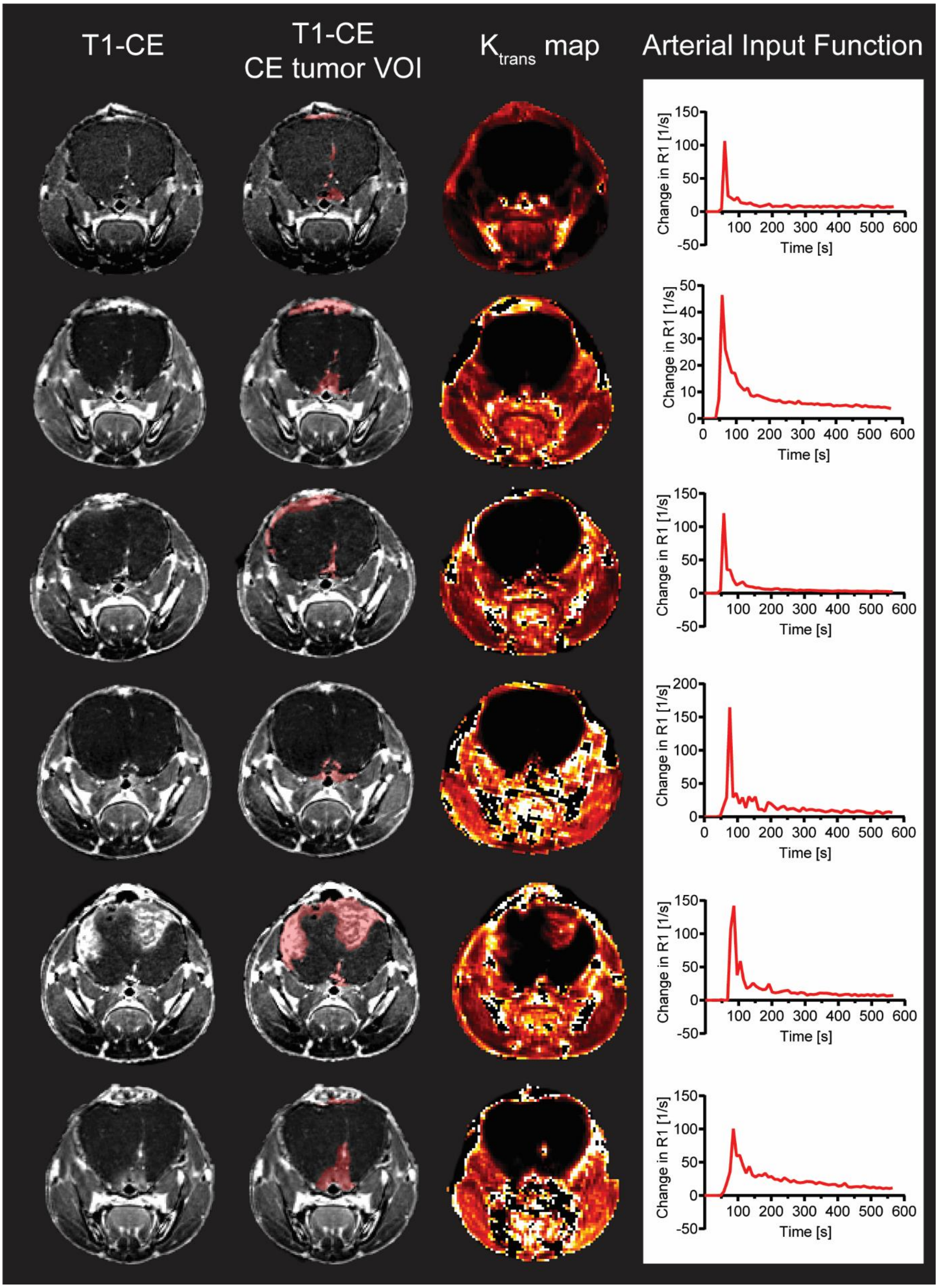

T1-CE
T1-CE
CE tumor VOI
K_{trans} map
Arterial Input Function
Change in R1 [1/s]
Time [s]
150
100
50
0
-50
100 200 300 400 500 600
50
40
30
20
10
0
0 100 200 300 400 500 600
200

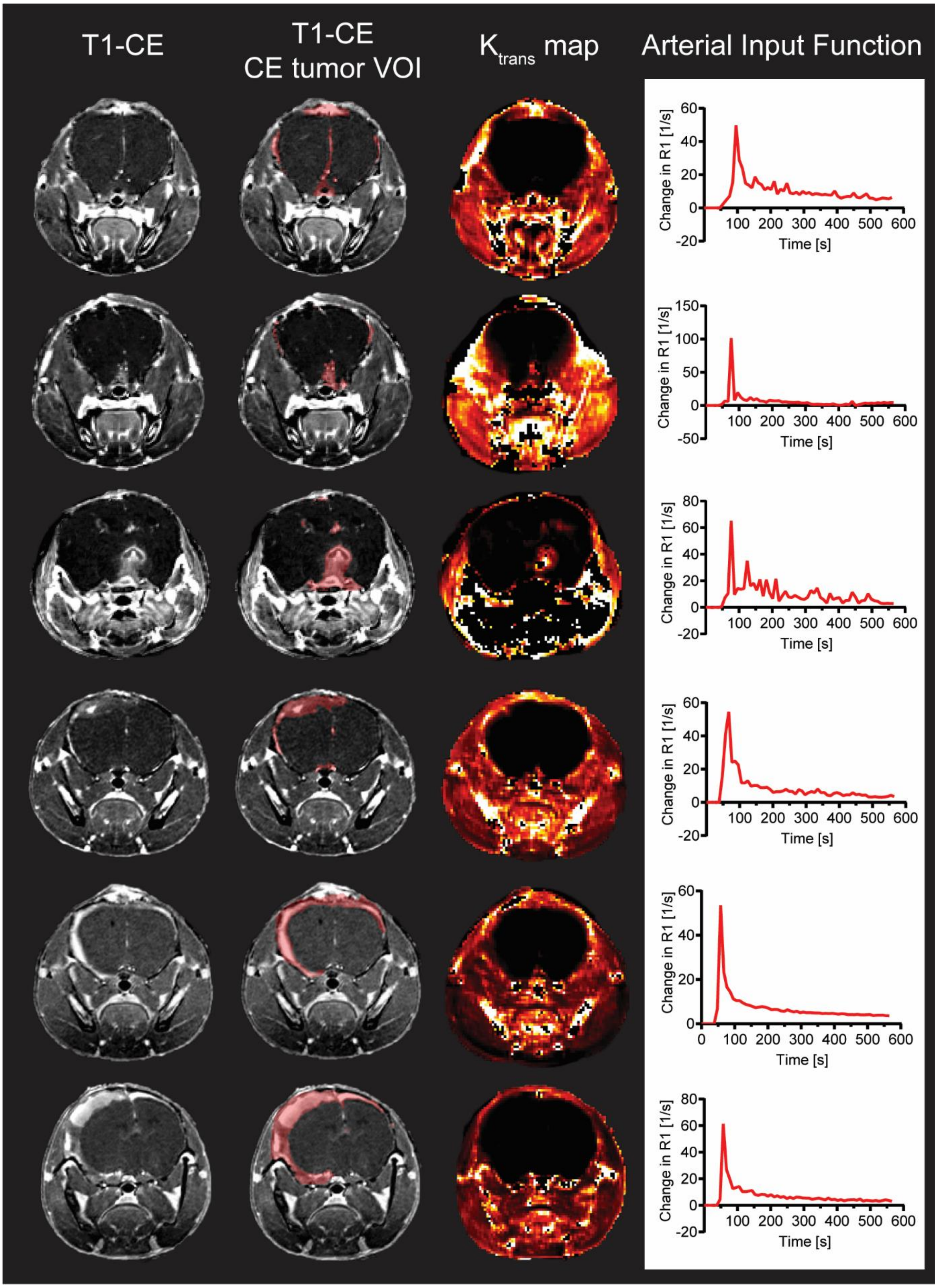
T1-CE
T1-CE
CE tumor VOI
K$_{trans}$ map
Arterial Input Function
Change in R1 [1/s]
Time [s]
0
100
200
300
400
500
600
-20
20
40
60
80
-50
50
150

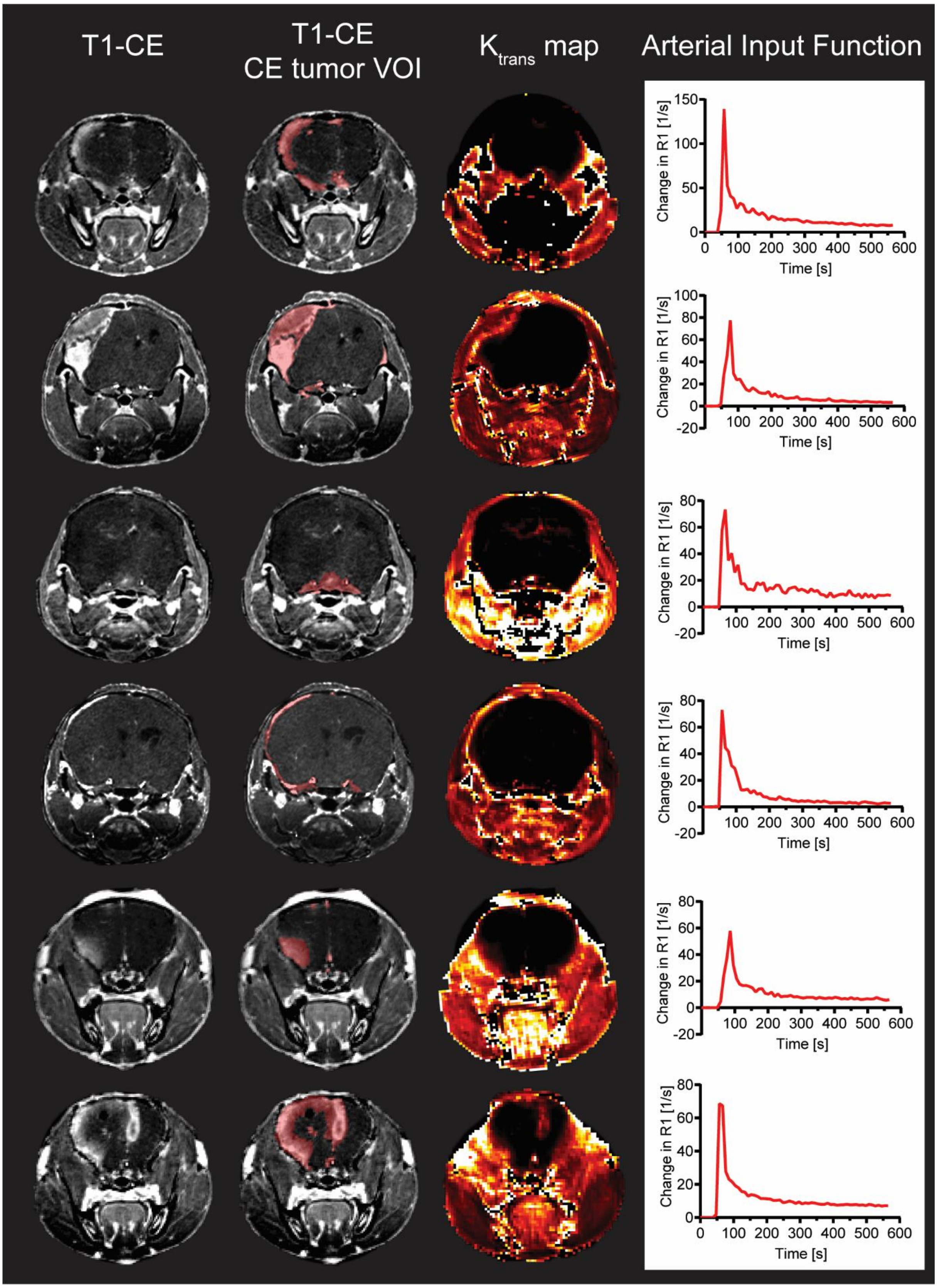

Figure S2. *DCE MRI modelling for K_{trans} maps generation in WK1 mouse model.* L-R: T1-CE, T1-CE with defined CE tumor VOI (red), K_{trans} map generated from kinetic modelling of DCE MRI data and arterial input function (AIF) selected for the generation for the K_{trans} map.

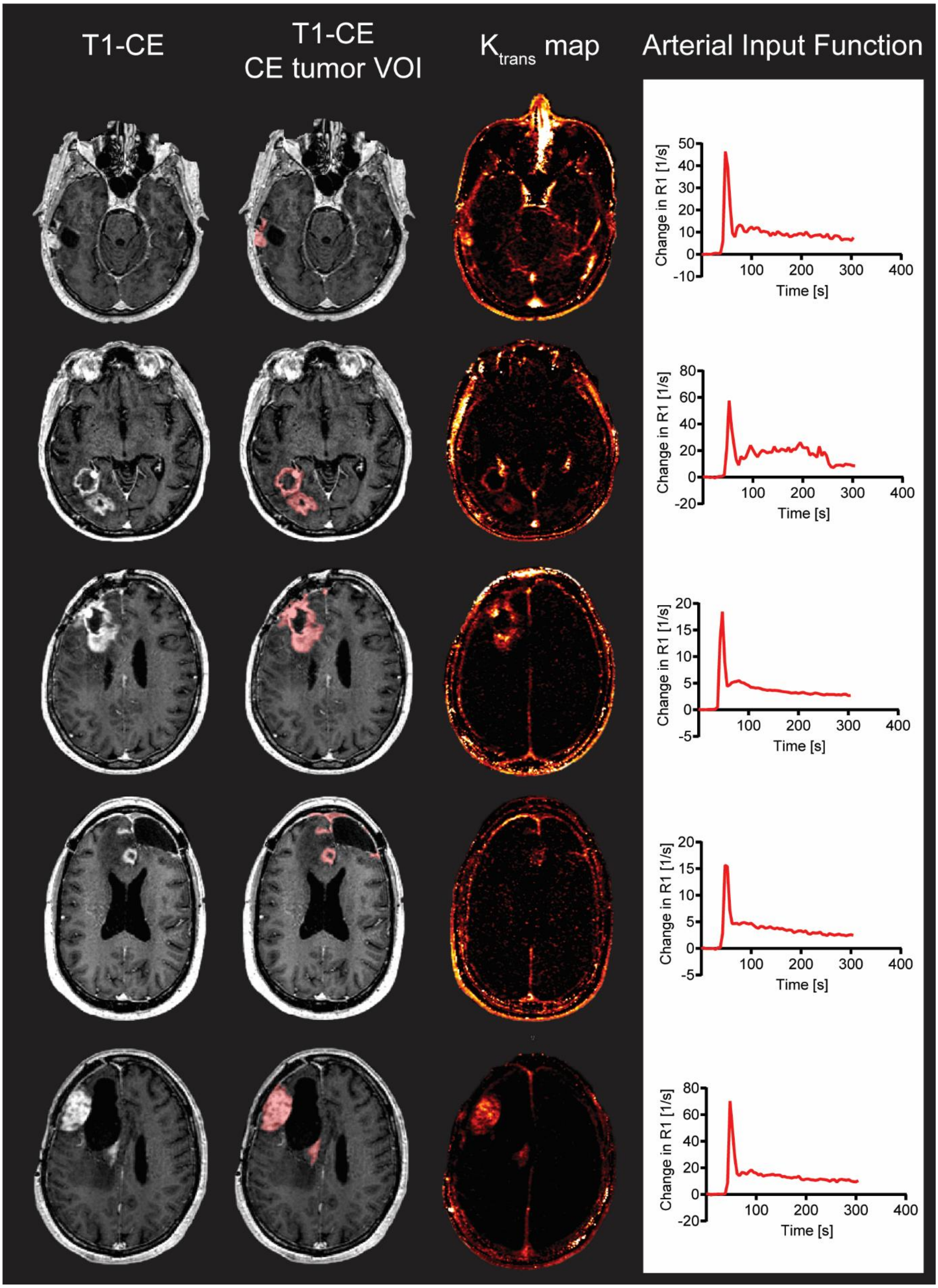
T1-CE
T1-CE
CE tumor VOI
K_{trans} map
Arterial Input Function
Change in R1 [1/s]
Time [s]

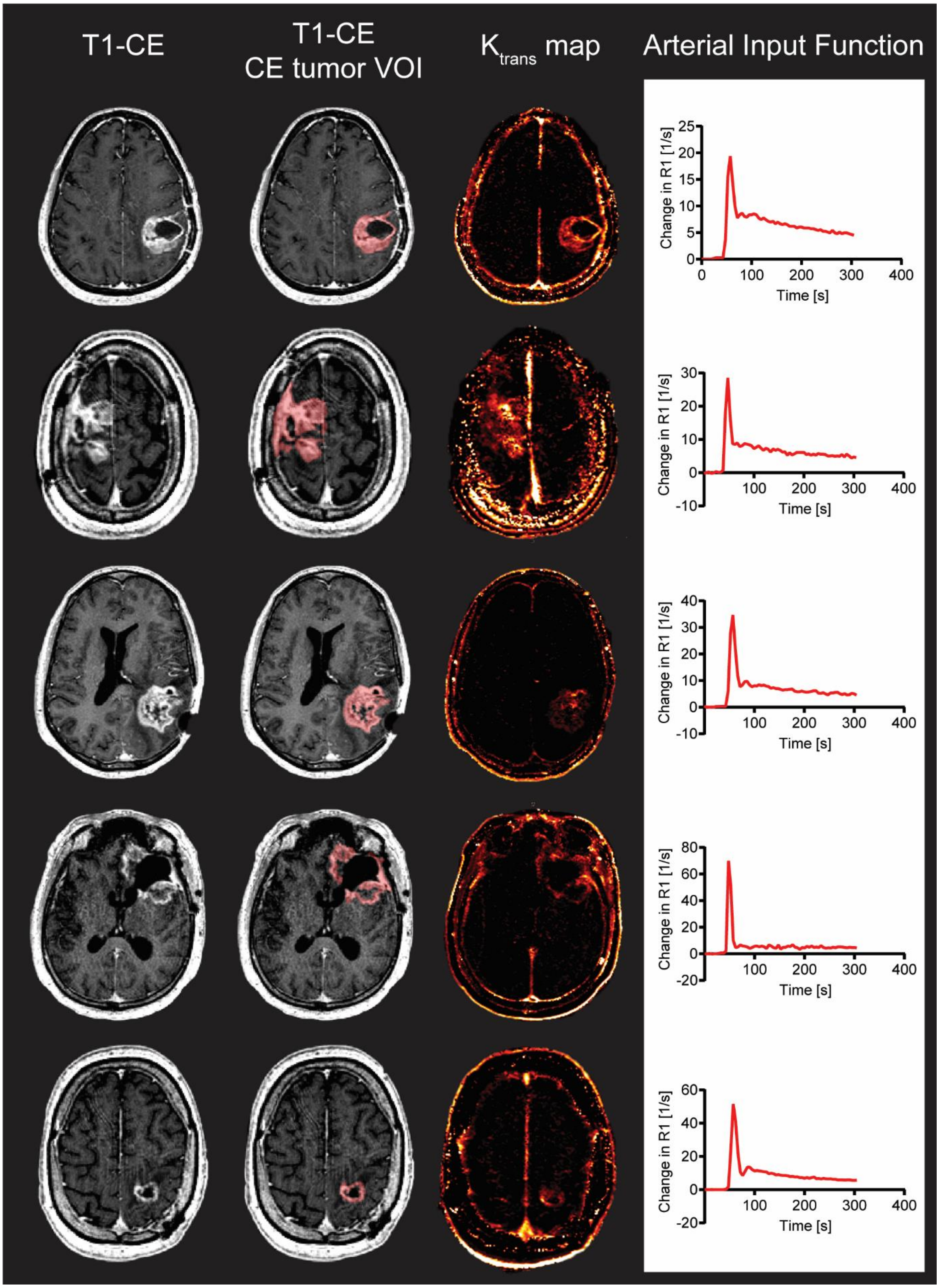
T1-CE
T1-CE
CE tumor VOI
K$_{trans}$ map
Arterial Input Function
Change in R1 [1/s]
Time [s]

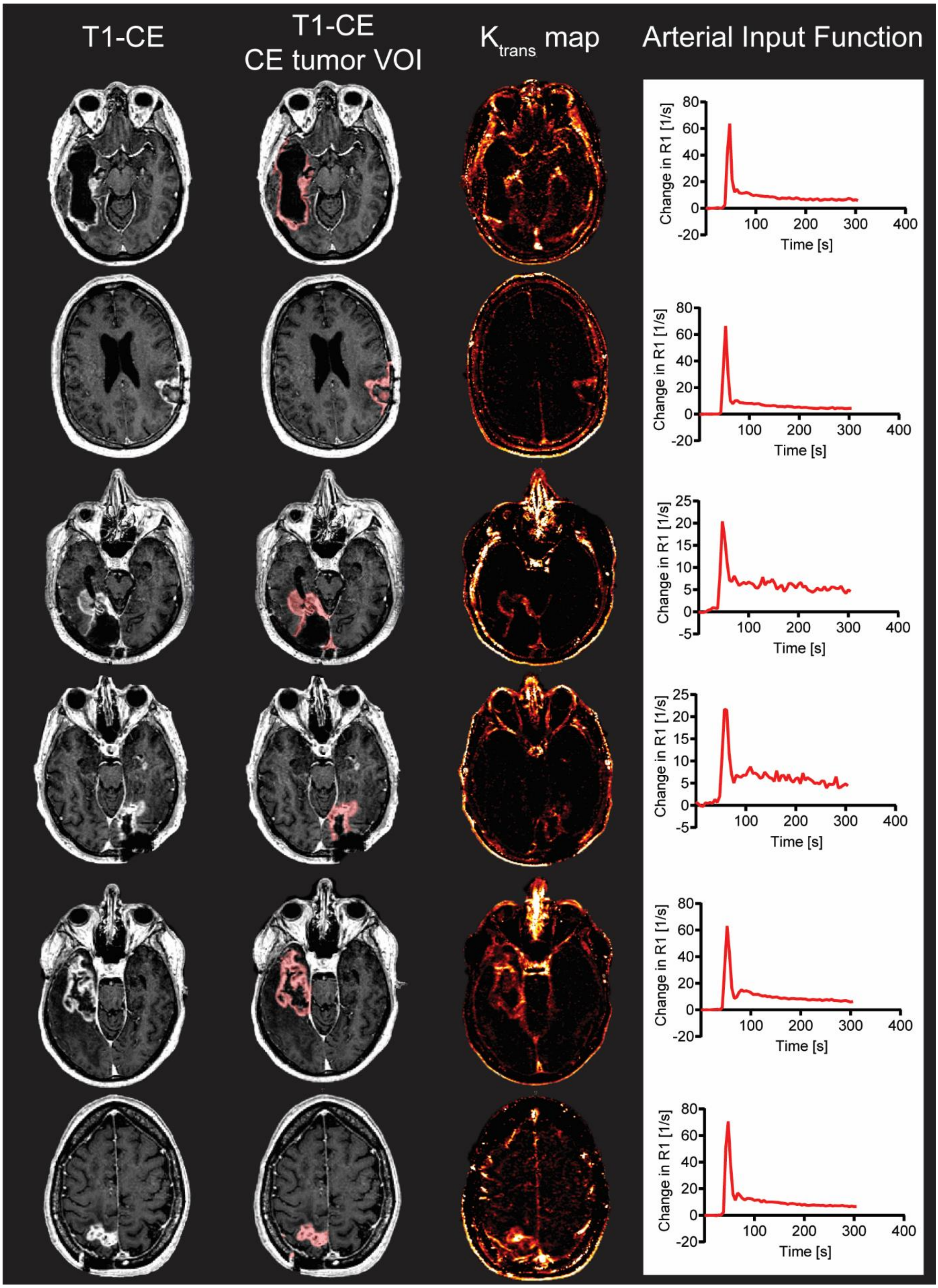

Figure S3. *DCE MRI modelling for K_{trans} maps generation in patient rHGG.* L-R: T1-CE, T1-CE with defined CE tumor VOI (red), K_{trans} map generated from kinetic modelling of DCE MRI data and arterial input function (AIF) selected for the generation for the K_{trans} map.

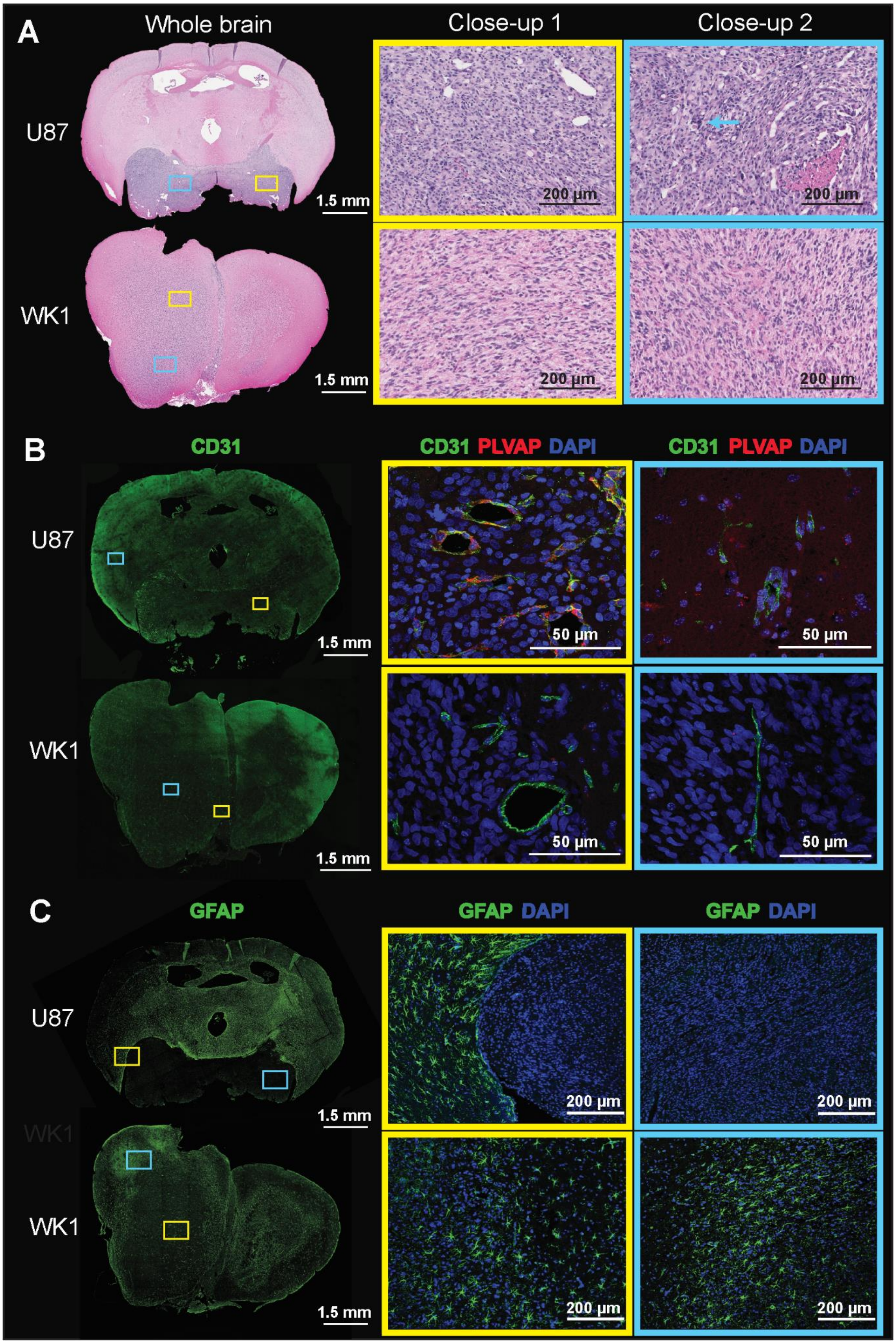

Figure S4. *Additional images of H&E and immunofluorescence stained brain sections.* The images show **A.** H&E, **B.** CD31/PLVAP/DAPI and **C.** GFAP staining of whole brain sections, and magnification of two areas (close-up 1 - yellow rectangle, close-up 2 - blue rectangle) of the U87 and WK1 mouse models.

Table S1. Values of mean K_{trans} and mean K_{ep} in the CE tumor VOI for each subject in the study.

Group	Subject ID	K_{trans} [min^{-1}]	K_{ep} [min^{-1}]
**U87**	U87_001	0.05	0.24
	U87_002	0.07	0.30
	U87_003	0.06	0.24
	U87_004	0.03	0.26
	U87_005	0.05	0.22
	U87_006	0.03	0.38
	U87_007	0.03	0.27
	U87_008	0.03	0.23
	U87_009	0.03	0.29
	U87_010	0.06	0.33
	U87_011	0.08	0.29
	U87_012	0.06	0.25
**WK1**	WK1_001	0.01	0.26
	WK1_002	0.05	0.16
	WK1_003	0.05	0.15
	WK1_004	0.03	0.16
	WK1_005	0.02	0.15
	WK1_006	0.02	0.20
	WK1_007	0.03	0.24
	WK1_008	0.04	0.12
	WK1_009	0.02	0.11
	WK1_010	0.05	0.27
	WK1_011	0.08	0.28
	WK1_012	0.03	0.15
	WK1_013	0.03	0.13
	WK1_014	0.03	0.23
	WK1_015	0.01	0.23
	WK1_016	0.01	0.13
	WK1_017	0.02	0.10
	WK1_018	0.03	0.19
**rHGG**	RIDER_001	0.03	0.35
	RIDER_002	0.01	0.22
	RIDER_003	0.05	0.24
	RIDER_004	0.04	0.23
	RIDER_005	0.02	0.29
	RIDER_006	0.03	0.30
	RIDER_007	0.02	0.25
	RIDER_008	0.02	0.20
	RIDER_009	0.03	0.27
	RIDER_010	0.02	0.17
	RIDER_011	0.02	0.30
	RIDER_012	0.02	0.18
	RIDER_013	0.03	0.38
	RIDER_014	0.02	0.32
	RIDER_015	0.02	0.18
	RIDER_016	0.03	0.20
